# Prenatal and postnatal exposure to acetaminophen in relation to autism spectrum and attention-deficit and hyperactivity symptoms in childhood: Meta-analysis in six European population-based cohorts

**DOI:** 10.1007/s10654-021-00754-4

**Published:** 2021-05-28

**Authors:** Silvia Alemany, Claudia Avella-García, Zeyan Liew, Raquel García-Esteban, Kosuke Inoue, Tim Cadman, Mònica López-Vicente, Llúcia González, Isolina Riaño Galán, Ainara Andiarena, Maribel Casas, Katerina Margetaki, Katrine Strandberg-Larsen, Deborah A. Lawlor, Hanan El Marroun, Henning Tiemeier, Carmen Iñiguez, Adonina Tardón, Loreto Santa-Marina, Jordi Júlvez, Daniela Porta, Leda Chatzi, Jordi Sunyer

**Affiliations:** 1 ISGlobal, Barcelona Institute for Global Health, C. Doctor Aiguader 88, 08003 Barcelona, Spain; 2 Universitat Pompeu Fabra (UPF), Barcelona, Spain; 3 CIBER Epidemiology and Public Health (CIBERESP), Madrid, Spain; 4 Hospital Sagrat Cor, Martorell, Spain; 5 Departmen of Environmental Health Sciences, Yale School of Public Health, New Haven, USA; 6 Yale Center for Perinatal, Pediatric, and Environmental Epidemiology, Yale School of Public Health, New Haven, USA; 7 Department of Epidemiology, Fielding School of Public Health, University of California, Los Angeles (UCLA), Los Angeles, USA; 8 MRC Integrative Epidemiology Unit (IEU) and School of Social and Community Medicine, University of Bristol, Bristol, UK; 9 Bristol Medical School, Population Health Science, Bristol, UK; 10 Department of Child and Adolescent Psychiatry, Erasmus MC–Sophia, Rotterdam, The Netherlands; 11 Epidemiology and Environmental Health Joint Research Unit, FISABIO-Universitat Jaume I-Universitat de València, Valencia, Spain; 12 Paediatrics, Hospital Universitario Central de Asturias, University of Oviedo and ISPA, Oviedo, Spain; 13 Faculty of Psychology, University of the Basque Country, Gipuzkoa, Spain; 14 Health Research Institute, Biodonostia, San Sebastian, Spain; 15 Department of Social Medicine, University of Crete, Crete, Greece; 16 Section for Epidemiology, Department of Public Health, University of Copenhagen, Copenhagen, Denmark; 17 Bristol NIHR Biomedical Research Centre, Bristol, UK; 18 Department of Pediatrics, University Medical Center Rotterdam, Erasmus MC, Rotterdam, The Netherlands; 19 Department of Psychology, Education and Child Studies, Erasmus School of Social and Behavioral Sciences, Erasmus University Rotterdam, Rotterdam, The Netherlands; 20 Department of Social and Behavioral Science, Harvard TH Chan School of Public Health, Boston, USA; 21 Department of Statistics and Computational Research, Universitat de València, València, Spain; 22 Health Research Institute of the Principality of Asturias (ISPA), IUOPA, University of Oviedo, Oviedo, Spain; 23 Health Research Institute, Biodonostia, San Sebastian, Spain; 24 Public Health Division of Gipuzkoa, Basque Government, Gipuzkoa, Spain; 25 Institut d’Investigació Sanitària Pere Virgili (IISPV), Hospital Universitari Sant Joan de Reus, Reus, Catalonia, Spain; 26 Department of Epidemiology, Lazio Regional Health Service, Rome, Italy; 27 Department of Preventive Medicine, University of Southern California, University Park Campus, Los Angeles, USA; 28 IMIM (Hospital del Mar Medical Research Institute), Barcelona, Spain

**Keywords:** Acetaminophen, Paracetamol, Pregnancy, Autism, Attention-deficit/hyperactivity disorder

## Abstract

The potential etiological role of early acetaminophen exposure on Autism Spectrum Conditions (ASC) and Attention-Deficit/Hyperactivity Disorder (ADHD) is inconclusive. We aimed to study this association in a collaborative study of six European population-based birth/child cohorts. A total of 73,881 mother–child pairs were included in the study. Prenatal and postnatal (up to 18 months) acetaminophen exposure was assessed through maternal questionnaires or interviews. ASC and ADHD symptoms were assessed at 4–12 years of age using validated instruments. Children were classified as having borderline/clinical symptoms using recommended cutoffs for each instrument. Hospital diagnoses were also available in one cohort. Analyses were adjusted for child and maternal characteristics along with indications for acetaminophen use. Adjusted cohort-specific effect estimates were combined using random-effects meta-analysis. The proportion of children having borderline/clinical symptoms ranged between 0.9 and 12.9% for ASC and between 1.2 and 12.2% for ADHD. Results indicated that children prenatally exposed to acetaminophen were 19% and 21% more likely to subsequently have borderline or clinical ASC (OR = 1.19, 95% CI 1.07–1.33) and ADHD symptoms (OR = 1.21, 95% CI 1.07–1.36) compared to non-exposed children. Boys and girls showed higher odds for ASC and ADHD symptoms after prenatal exposure, though these associations were slightly stronger among boys. Postnatal exposure to acetaminophen was not associated with ASC or ADHD symptoms. These results replicate previous work and support providing clear information to pregnant women and their partners about potential long-term risks of acetaminophen use.

**Supplementary Information:**

The online version contains supplementary material available at 10.1007/s10654-021-00754-4.

## Introduction

Acetaminophen (or paracetamol) is used by 46–56% of pregnant women in developed countries [1–3]. Whilst acetaminophen is considered the safest analgesic/antipyretic for pregnant women and children, mounting evidence has linked prenatal acetaminophen exposure to worse cognitive performance [4–6], more behavioural problems [1, 7, 8], Autism Spectrum Conditions (ASC) [6, 9] and Attention-Deficit/Hyperactivity Disorder (ADHD) symptoms [1, 3, 6, 10]. Recently, detection of acetaminophen in meconium has associated with increased odds of ADHD and altered frontoparietal connectivity at ages 9–10 years [11].

Two meta-analyses have investigated the link between prenatal acetaminophen use and ASC and ADHD symptoms [12, 13]. The first meta-analysis included seven cohort studies (N = 132,738) and reported risk increases of 19% for ASC and 34% for ADHD [12]. The second meta-analysis focused on ADHD, included eight cohort studies (N = 244,940) and concluded that exposed children had a 25% increased risk of developing ADHD symptoms [13]. However, the methods and instruments used to assess the outcome in the studies included in the meta-analysis are highly heterogeneous [14, 15]. Another source of heterogeneity among these studies regards the statistical approach and confounders included [14]. Furthermore, these studies did not address relevant unsolved questions regarding the link between early acetaminophen exposure and ASC and ADHD symptoms.

First, it is unclear whether girls or boys are differentially affected by acetaminophen exposure. A recent study examining biomarkers of prenatal acetaminophen exposure among 996 mother–child pairs observed higher odds for ASC among boys and higher odds for ADHD among girls. [16] This is partially in agreement with a population-based study conducted in INfancia y Medio Ambiente (INMA) cohort representing 2644 mother–child pairs, where prenatal acetaminophen exposure was positively associated with ASC symptoms only among boys. [6] However, two studies including 64,322 mothers enrolled in the Danish National Birth Cohort (DNBC), reported slightly higher estimates among girls for ADHD and ASC without hyperkinetic symptoms [1, 9]. Second, the abovementioned studies on INMA cohort [6] and acetaminophen biomarkers [16] observed positive associations with both ASC and ADHD symptoms. This contrasts with the study conducted in the DNBC cohort where prenatal exposure was linked to ASC when accompanied by hyperkinetic symptoms [9]. Finally, another gap in research relates to postnatal exposure. An ecological study found positive correlations between indicators of both prenatal and postnatal acetaminophen exposures and ASC prevalence [17]. To our knowledge, postnatal associations have not been examined in prospective cohort studies.

In the current study, we aim to examine the association between early acetaminophen exposure and ADHD and ASC symptoms and hospital diagnosis. Furthermore, we also examined postnatal exposure and we present sex-stratified results. To reduce heterogeneity, we used a common set of confounders, the same statistical approach and harmonized the exposure and outcomes measurements across cohorts.

## Methods

### Sample

We included six European population-based birth cohorts: Avon Longitudinal Study of Parents and Children (ALSPAC), DNBC, Gene and Environment: Prospective Study on Infancy in Italy (GASPII), the Generation R Study, INMA (including four subcohorts), and the Mother–Child Cohort in Crete (RHEA) (Methods S1). Mother–child pairs were recruited from 1991 through 2008. A total of 73,881 children with available data on either prenatal or postnatal exposure to acetaminophen and at least one outcome (ASC or ADHD symptoms) and main covariates were included (60.5% of the children recruited at baseline).

Informed consent was obtained from all participants in each cohort and ethical approval was obtained from the local authorized institutional review boards.

### Exposure

Regarding prenatal acetaminophen exposure, mothers were interviewed two (INMA, RHEA), three (GASPII) or four (DNBC) times during pregnancy using standardized questionnaires. At each interview, mothers were asked if they had taken medications from the month before becoming pregnant (GASPII; INMA) or beginning of pregnancy (RHEA) through delivery. In ALSPAC and the Generation R Study, mothers completed questionnaires two (ALSPAC) or three (the Generation R Study) times during pregnancy reporting acetaminophen use from the month before becoming pregnant to gestational week 32 of pregnancy. In GASPII, mothers were interviewed at birth and provided retrospective information on acetaminophen use at each trimester. Mothers were classified as ever exposed if they reported having taken any dose of acetaminophen in the defined prenatal exposure period; otherwise they were classified as non-exposed (More details in Table 1 and Supplemental Materials (S) Methods S2).

**Table 1:** Assessment of exposures

Cohort study	Pregnancy period	N^a^	Prenatal			Postnatal		
			Instrument	N Exposed	% Exposed	Instrument	N Exposed	% Exposed
ALSPAC	1991–1992	6200	Maternal questionniare completed at 18 and 32 weeks of pregnancy	2425	39.1	Maternal questionniare completed when children were 12 months old	375	6.0
DNBC	1996–2002	61,430	Self-reported study enrolment form and in three computer-assisted telephone interviews (12th and 30th week, and 6-month postpartum)	34,584	56.3	Computer-assisted telephone interviews with mothers when children were 6 months and 18 months old	4735	7.7
GASPII	2003–2004	489	Interview with mothers at first, second and third trimesters of pregnancy	153	31.3	Interview with moths when children were 6 and 15 months old	454	92.8
Generation R	2001–2005	3904	Maternal questionniare completed at 12, 20 and 30 weeks of pregnancy	1150	29.5	Maternal questionniare completed when children were 12 months old	2526	64.7
INMA	2004–2008	1513	Interview with mothers at weeks 12 and 32 of pregnancy	775	51.2	Maternal questionnaires when children were 6, 14 or 18 months old (depending on subcohort)	750^b^	90.8 ^b^
RHEA	2007–2008	345	Interview with mothers at weeks 12 and 30 of pregnancy	49	14.2	Interview with mothers when children were 9 months old	306	88.7

^a^Number of children with data available on early acetaminophen exposure (prenatal or postnatal), at least one outcome (ASC or ADHD symptoms) and main covariates

^b^Number of children with data available for postnatal exposure, at lest one outcome and main covariates is 829

Regarding postnatal acetaminophen exposure, mothers were interviewed (DNBC, GASP, RHEA) or completed questionnaires (INMA) about medication use in their children one (RHEA) or two times (The Generation R Study, GASP, INMA, DNBC) in the first 18 months of life of the child. At each interview, mothers were asked if they had given any medication including acetaminophen, to their child. Children were classified as postnatally exposed to acetaminophen if they had taken any dose of acetaminophen at any time up to 18 months of life. Otherwise, they were considered non-exposed (Table 1, Methods S3).

### Outcomes

ASC and ADHD symptoms were assessed using validated parent-reported questionnaires or linked hospital records. Autistic symptoms were assessed using the Development And Well-Being Assessment (DAWBA) [18] (ALSPAC), the Pervasive Developmental Problems (PDP) subscale of the Child Behaviour Checklist for Toddlers (CBCL1½–5) [19] (GASPII and The Generation R Study), the Childhood Autism Spectrum Test (CAST) [20] (INMA) and an ASC scale derived from the CBCL for 6–18 (CBCL6–18) [21] (RHEA). ADHD symptoms were assessed using the Development and Well-Being Assessment (DAWBA) [18] (ALSPAC); the Conner’s Parent Rating Scale Revised short form (CPRS-R:S) [22] (The Generation R Study), the Hyperactivity/Inattention subscale of the Strengths and Difficulties Questionnaire (SDQ) [23] (DNBC), the Attention Deficit and Hyperactivity problems subscale of the CBCL1½-5 and CBCL6/18 (GASPII and RHEA), and the ADHD Criteria of DSM-IV (DSM-ADHD Questionnaire) [24] (INMA). Higher scores indicate more symptoms.

In addition to the SDQ-Hyperactivity/Inattention subscale, in the DNBC cohort, diagnoses of ASC and ADHD from national hospital register were also available [1].

To harmonize the continuous scores on ASC and ADHD symptoms, we used questionnaire-specific validated cut-offs to yield proxies for ASC and ADHD symptoms within the borderline/clinical range. Specifically, for the DSM-ADHD Questionnaire scores, we applied established cut-offs consisting of 6 or more symptoms of inattention or hyperactivity to yield proxies for borderline/clinical ADHD symptoms [24]. For CBCL1½-5 and CBCL6/18 scales, we used the recommended cut-off to classify children with borderline/clinical symptoms corresponding to scores above 93rd percentile [25, 26]. Of note, since no specific cut-offs have been established for the ASC-CBCL scale [21], we applied the recommended cut-off abovementioned for the CBCL6/18 scales.^25^ The ADHD index of the CSRS-R:S has a cut-off for elevated T-scores (65–69) that we used to classify children as presenting borderline/clinical symptoms (T-scores > 65) [22]. The DAWBA is based on diagnostic criteria (ICD-10 and DSM-IV) and focuses on anxiety disorders, depressive disorders, ADHD and conduct disorders. A clinical diagnostic rating is informed by triangulation of these three sources [27]. In the case of SDQ, abnormal scores in emotional problems and in peer problems or prosocial behaviour subscales were used to capture ASC borderline/clinical symptoms and abnormal scores in the SDQ hyperactivity/inattention and impact subscales were used to capture ADHD borderline/clinical symptoms [28]. Finally, for SCDC scores, the cut-off of 8 to detect probable cases was applied to classify children as having borderline/clinical ASC symptoms [29]. Further details on outcomes measures and cut-offs applied can be found in Table 2 and Methods S4–S5. A summary of the psychometric properties of the instruments used to assess the outcomes can be found in Table S1.

**Table 2:** Assessment of behavioral outcomes

Cohort Study	N^a^	ASC symptoms					ADHD symptoms				
		Instrument	Age (years)	Informant	Individuals within borderline/clinical range		Instrument	Age (years)	Informant	Individuals within borderline/clinical range	
					n	%				n	%
ALSPAC	6200	SCDC	7	Parents	414	6.68	DAWBA	7	Mothers	120	1.9
DNBC	61,430	SDQ	7	Mothers	573	0.9	SDQ	7	Mothers	729	1.2
		Hospital diagnosis	12.8	Medical doctor	970	1.6	Hospital diagnosis	12.8	Medical doctor	1289	2.1
Generation R	3904	CBCL 1/2–5	6	Parents	299	7.7	CPRS-R:S	8	Parents	298	7.6
GASPII	489	CBCL 1/2–5	4	Parents	63	12.9	CBCL 1.5/5	4	Parents	50	10.2
INMA	1513	CAST	4.5	Parents	99	6.5	ADHD-DSM-IV	4.5	Teachers	175	11.6
RHEA	345	CBCL 6/18	6	Parents	33	9.6	CBCL 6/18	6	Parents	21	12.2

*CAST* childhood asperger syndrome test, *CBCL* child behaviour checklist,*CPRS-R:S* conner's parent rating scale revised short form, *DAWBA* development and well-being assessment, *PDP* pervasive developmental problems, *SDQ* strenghts and difficulties questionnaire, *SCDC* social communication disorders checklist

^a^Number of children with data available on early acetaminophen exposure (prenatal or postnatal), at least one outcome (ASC or ADHD symptoms) and main covariates

### Covariates

Potential confounding variables were selected a priori prioritizing availability and consistency. Covariates were factors previously associated with ASC, ADHD and acetaminophen exposure, and include maternal and child characteristics. Maternal characteristics included age at delivery (years), education (low, medium, high), pre-pregnancy body-mass index (BMI), alcohol (yes/no), smoking (yes/no) and mental health problems (yes/no) during pregnancy, age at birth (years) and parity (nulliparous, > 1 and > 2), maternal fever (yes/no) and infections (yes/no) during pregnancy. Maternal education was not provided by the DNBC and analyses were adjusted by maternal socio-occupational status based on job titles instead. Child characteristics included sex, age at behavioural assessment (years), cold (yes/no) and respiratory infections (yes/no) in the first 2 years of life. Maternal characteristics were collected during pregnancy except for mental health in INMA, which was collected when children were around 5 years. Child characteristics were collected in the first 18 months of life. Two cohorts adjusted their analysis for cohort-specific covariates. Specifically, analysis in Generation R Study were also adjusted by ethnicity (Dutch, non-Dutch/other western, non-western) and analysis in INMA were also adjusted by subcohort (INMA-Asturias, INMA-Gipuzkoa, INMA-Sabadell and INMA-Valencia).

### Statistical analysis

All analyses were performed following the same protocol. We used logistic regression models to assess the association between prenatal and postnatal acetaminophen exposure and ASC or ADHD symptoms (as binary outcomes) within the borderline/clinical range. Separate models were conducted for prenatal and postnatal exposure, and for ASC and ADHD symptoms. Main models were adjusted for the abovementioned covariates and stratified by child sex.

Cohort-specific analyses were undertaken at each study centre and pooled using random-effects meta-analysis. Random effects models, which account for unexplained heterogeneity, assume that the effect size varies across the studies because of real differences in the exposure effect and sampling variability [30]. Between-study heterogeneity was assessed using Cochran’s *Q* test and the *I*^*2*^ statistic.

Since the cohorts included herein differ in the length of follow-up, we performed meta-regression including mean age of the child at outcome assessment as moderator. These analyses allow us to examine potential changes in the association between acetaminophen exposure and ASC and ADHD symptoms over time.

Sensitivity analyses included: (a) testing the associations with hospital diagnosis of ASC and ADHD available in the DNBC cohort, (b) testing the associations with ASC symptoms excluding ADHD cases, (c) meta-analyses leaving out one cohort at a time to determine the influence of each cohort, and (d) additional adjustment for gestational age, birthweight (grams), maternal chronic diseases -except psychiatric diseases- (yes/no), maternal use of other drugs (yes/no) and maternal folic acid use (yes/no).

Analyses were performed using R 3.5.1 (https://www.r-project.org/). Logistic regression models to estimate odds ratio (OR) and 95% confidence interval (CI) were fitted using *finalfit* package (https://finalfit.org/index.html). Meta-analyses were conducted using *metafor* package [31].

## Results

Across studies between 14 and 56% of mothers reported acetaminophen use in the prenatal period, with the RHEA cohort having the lowest proportion and DNBC the highest. A wider range of child postnatal acetaminophen exposure was reported varying from 6% in ALSPAC to 92.8% in GASPII (Table 1).

The total number of children having ASC or ADHD symptoms within the borderline/clinical range was 1,481 (2.1%) and 1,393 (2%), respectively (Table 2). Across cohorts, we observed large variations in the proportion of children presenting borderline/clinical symptoms with 0.9% and 12.9% presenting ASC symptoms and between 1.2 and 12.2% presenting ADHD symptoms.

Table 3 shows the distributions of maternal and child characteristics.

**Table 3:** Distribution of the child and maternal characteristics

Cohort	N^a^	Sex (Female), %	Child age (ASC)		Child age (ADHD)		Maternal age		Parity, %			Maternal education^b^, %			Maternal BMI		Maternal Alcohol (Yes), %	Maternal Smoking (Yes), %	Maternal Mental Health Problems (Yes), %
			M	SD	M	SD	M	SD	0	1	> 2	Low	Medium	High	M	SD			
ALSPAC	6200	50.8	7.7	0.1	7.7	0.1	28.9	4.5	45.8	36.5	17.7	20.7	61.9	17.5	22.8	3.6	79.8	19.7	8.5
DNBC	61,430	51.0	7.2	1.6	7.2	1.6	30.5	4.2	45.7	37.5	16.8	3.6	29.1	67.3	23.7	4.4	70.6	25.7	7.9
			12.8^c^	1.6^c^	12.8^c^	1.6^c^													
GASPII	489	51.5	4.1	0.3	4.1	0.3	33.7	4.3	57.5	37.4	5.1	10.2	51.7	38.0	22.2	3.4	19.0	41.1	4.7
Generation R	3904	49.3	6.0	0.5	8.2	0.2	30.9	4.7	59.6	30.1	10.3	6.0	40.6	53.4	23.4	4.1	59.4	23.0	8.1
INMA	1513	51.6	4.9	0.6	4.9	0.6	32.3	4.1	57.3	36.6	6.1	20.9	41.0	38.1	23.6	4.2	9.1	29.8	17.4
RHEA	345	59.7	6.6	0.3	6.6	0.3	30.8	4.7	44.6	38.8	16.5	13.6	50.4	35.9	24.9	5.1	30.1	33.0	1.2

^a^Number of children with data available on early acetaminophen exposure, main covariates and at least one of the outcomes studied (autistic or ADHD symptoms)

^b^Maternal education was not provided in DNBC. Parental socio-occupational status based on the highest of maternal or paternal education and occupation was used instead

^c^Parent-reported questionnaire (SDQ)/Hospital diagnoses

Children with ASC symptoms within the borderline/clinical range were more likely to be males and their mothers were more likely to be younger, have lower educational level and report alcohol consumption and mental health problems during pregnancy compared to children not in the borderline/clinical range (Table S2). Children with ADHD symptoms within the borderline/clinical range were also more likely to be males and have a higher proportion of nulliparous mothers, who smoked during pregnancy and experienced mental health problems during pregnancy compared to children not in the borderline/clinical range (Table S3).

Children prenatally exposed to acetaminophen were overall more likely to have older and non-nulliparous mothers with higher education levels and higher pre-pregnancy BMI who report alcohol consumption, smoking and mental health problems during pregnancy (Table S4). Children postnatally exposed to acetaminophen were more likely to have nulliparous mothers, who have higher education levels and report alcohol consumptions during pregnancy (Table S5).

### Prenatal acetaminophen exposure and ASC symptoms

**Fig. 1:**
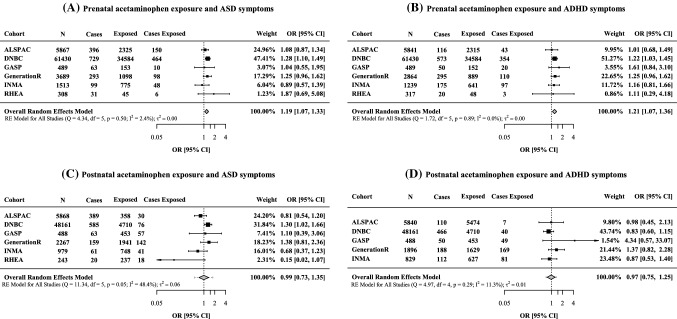
Associations between early acetaminophen exposure and autistic autism spectrum condition (ASC) (**a**, **c**) and attention-deficit and hyperactivity (ADHD) symptoms (**b**, **d**) within the borderline/clinical range. Associations for prenatal (**a**, **b**) and postnatal (**c**, **d**) exposure are shown. Symptoms were assessed using parent and teacher reported questionnaires in all cohorts. Odds Ratio (OR) and 95% confidence intervals (CI) by cohort and overall estimate obtained from random-effects meta-analysis. Models were adjusted for maternal characteristics (education, age at delivery, pre-pregnancy body mass index, prenatal smoking, mental health during pregnancy, parity and alcohol consumption, fever and infections during pregnancy) and child’s characteristics (sex, age at the behavioural assessment). Postnatal models were further adjusted by child’s cold or respiratory infections. Models on postnatal exposure and ADHD symptoms were not possible to conduct in RHEA cohort (limited sample size)

Children prenatally exposed to acetaminophen were 19% more likely to subsequently have ASC symptoms within the borderline/clinical range than non-exposed children (OR = 1.19, 95% CI 1.07–1.33) (Fig. 1). Similar results were observed using hospital diagnosis in DNBC cohort (OR = 1.16, 95% CI 1.05–1.29) (Fig. S1). When stratifying by sex, prenatal acetaminophen was associated with ASC symptoms among boys (OR = 1.28, 95% CI 1.12, 1.46) and to a lesser extent among girls (OR = 1.06, 95% CI 0.82, 1.36) (Table 4), though there was no statistical evidence of a difference between boys and girls (*P*_interaction_ = 0.188). When using hospital diagnosis in DNBC, a similar effect size was observed in boys (OR = 1.14, 95% CI 1.00, 1.29) and girls (OR = 1.15, 95% CI 0.78, 1.71) (Table S6). Excluding ADHD cases, prenatal acetaminophen exposure was associated with ASC symptoms based on questionnaires in all cohorts (OR = 1.16, 95% CI 1.01, 1.43) (Table S7). Additional adjustment for other confounders did not change the results meaningfully (Table S8). In leave-one-out analyses results, the association was attenuated but remained positive when omitting DNBC (OR = 1.12, 95% CI 0.96, 1.30 while no changes were observed when removing each of the other cohorts (Table S9).

**Table 4:** Adjusted associations between prenatal and early postnatal acetaminophen exposure and borderline/clinical autistic spectrum (ASC) symptoms and attention-deficit and hyperactivity (ADHD) symptoms in boys and girls

			Boys						Girls				
Exposure period	Outcome	n^a^	N^b^	OR (95% CI)^c^	*I*^*2*^ (%)	Q-value	P-heter	n^a^	N^b^	OR (95% CI)^c^	*I*^*2*^ (%)	Q-value	P-heter
Prenatal	ASC symptoms	6	37,368	1.28 (1.12, 1.46)	0.02	5.747	0.332	5	35,073	1.06 (0.82, 1.36)	31.39	7.781	0.101
	ADHD symptoms	6	30,031	1.23 (1.05, 1.44)	0.00	1.848	0.887	5	35,265	1.18 (0.97, 1.44)	0.00	0.996	0.910
Postnatal	ASC symptoms	6	29,484	1.16 (0.90, 1.48)	7.74	12.915	0.024	5	28,520	0.87 (0.55, 1.37)	37.3	6.272	0.179
	ADHD symptoms	6	28,791	1.10 (0.70, 1.71)	43.08	5.825	0.124	5	28,172	0.83 (0.58, 1.19)	0.00	1.330	0.856

*I*^*2*^ percentage of the total variability due to between-study heterogeneity, *p*-*Heter p*-value of heterogeneity using the Cochran’s Q test

^a^Number of cohorts included in the meta-analysis

^b^Number of children included in the meta-analysis

^c^Odds ratios and 95% confidence intervals were estimated by random-effects meta-analysis. Models were adjusted for maternal characteristics (education, age at delivery, pre-pregnancy body mass index, prenatal smoking, mental health during pregnancy, parity and alcohol consumption, fever and infections during pregnancy) and child’s characteristics (sex, age at the behavioral assessment). Postnatal models were further adjusted by child’s cold or respiratory infections

Substantial between-study heterogeneity was only observed when examining this association among girls using hospital diagnosis in DNBC (*I*^*2*^ = 68.37%) (Table S6).

Meta-regression analyses showed that children’s age at outcome assessment did not modified the association between prenatal exposure to acetaminophen and ASC symptoms (*P*-value = 0.923).

### Prenatal acetaminophen exposure and ADHD symptoms

The odds of developing ADHD symptoms within the borderline/clinical range were 21% higher among children prenatally exposed to acetaminophen compared to non-exposed children (OR = 1.21, 95% CI 1.07–1.36) (Fig. 1). Prenatal acetaminophen use was associated with ADHD symptoms in boys (OR = 1.23, 95% CI 1.05, 1.44) and to a similar extent in girls (OR = 1.18, 95% CI 0.97, 1.44) (*P*_interaction_ = 0.747) (Table 4). In sensitivity analysis, we observed positive associations using hospital diagnoses in DNBC (OR = 1.30, 95% CI 1.14, 1.48) (Fig. S1) and consistent results in boys (OR = 1.31, 95% CI 1.15, 1.49) and girls (OR = 1.39, 95% CI 1.15, 1.68) (Table S6). Results were also similar with additional adjustment for confounders (Table S8) or in leave-one-out analysis (Table S9). There was no strong evidence of between-study heterogeneity (Fig. 1, Table 4, Fig. S2, Tables S6–S9).

Meta-regression analyses showed that children’s age at outcome assessment did not modified the association between prenatal exposure to acetaminophen and ADHD symptoms (*P*-value = 0.531).

### Postnatal acetaminophen exposure and ASC symptoms

No association was found between postnatal acetaminophen exposure and ASC (OR = 0.99, 95% CI 0.73–1.35) (Fig. 1). Stratified results by sex were also close to the null for both boys and girls (Table 4, Table S4). Similar results were observed when using hospital diagnosis in DNBC (Fig. 1S). Results were also close to the null when excluding ADHD cases (Table S5) and with additional adjustment for other confounders (Table S6). There was evidence of between-study study heterogeneity in main results (*I*^*2*^ = 48.4) (Fig. 1).

Meta-regression analyses showed that children’s age at outcome assessment did not modified the association between postnatal exposure to acetaminophen and ASC symptoms (*P*-value = 0.975).

### Postnatal acetaminophen exposure and ADHD symptoms

No association was found between postnatal acetaminophen exposure and ADHD symptoms (OR = 0.97, 95% CI 0.75–1.25) (Fig. 1). Associations were also null when stratifying by sex (Table 4, Table S6). Similar results were observed in sensitivity analysis using hospital diagnosis in DNBC (Fig. S1), with additional adjustment for other confounders (Table S8) and in leave-one-out analysis (Table S10). There was no strong evidence of marked between-study heterogeneity (Fig. 1, Fig S1, Table 4, Tables S6–S10).

Meta-regression analyses showed that children’s age at outcome assessment did not modified the association between postnatal exposure to acetaminophen and ADHD symptoms (*P*-value = 0.765).

## Discussion

The results of our meta-analysis representing more than 70,000 children of six European population-based birth/child cohorts indicated that children prenatally exposed to acetaminophen were 19% and 21% more likely to subsequently have ASC and ADHD symptoms within the borderline/clinical range, respectively, compared with non-exposed children. The association with ASC was attenuated after omitting the largest cohort but remained positive. When stratifying by sex, these associations were slightly stronger among boys compared to girls but positive associations with effect sizes of similar magnitude were observed in both strata, especially in the case of ADHD. Postnatal exposure to acetaminophen was not associated with either of the outcome, thought there was evidence of between-study heterogeneity for the association with ASC symptoms.

The most consistent pattern of results was observed for the association between prenatal acetaminophen exposure and ADHD symptoms. The positive associations were observed in all the cohorts and of similar magnitude regardless of the cohort excluded in the leave-one-out analysis. This finding is in agreement with previous meta-analysis which reported likelihood increases of 25% and 34% for ADHD in relation to prenatal acetaminophen exposure [12, 13]. Our findings are consistent with previous single cohort studies conducted in ALSPAC [7], DNBC [1, 9] and INMA [6] cohorts, which were included in our meta-analysis. Despite the overlap of samples included, this agreement supports the robustness of the findings since analytical strategies and outcome definitions were harmonized for the present meta-analysis.

The association between prenatal acetaminophen use and ASC symptoms was consistently positive even after omitting the largest cohort. Previous findings in DNBC only found this association in ASC cases with hyperactive symptoms [9], however, in our meta-analysis the association remained after excluding ADHD cases. Overall our findings provide support for the association between prenatal acetaminophen and ASC symptoms in line with a previous meta-analysis [12].

Associations between prenatal acetaminophen and ASC and ADHD symptoms were consistently positive for both boys and girls albeit slightly stronger among boys, with near identical odds ratios for hospital diagnosed cases in DNBC. Importantly, we found no evidence for statistical interaction between child sex and prenatal acetaminophen exposure and either ASC or ADHD symptoms. This contrasts with previous findings in single cohort studies reporting sex differences in the association between prenatal acetaminophen and ASC [6, 9, 16] and ADHD [8, 16]. Our findings suggest that differential sex effects of acetaminophen on ASC and ADHD symptoms, if any, are modest and may be dependent on the number of cases, outcome definition and assessment.

Associations between postnatal acetaminophen exposure and both ASC and ADHD symptoms were close to the null and different directions of associations around the null were observed across cohorts. Heterogeneity was high in the case of ASC, with about 50% of the variation in odds of ASC symptoms being explained by between study differences. The high prevalence of the exposure to acetaminophen when combining pre- and post-natal across studies made it impossible to explore cumulative effects combining prenatal and postnatal exposures. Previous studies examining postnatal acetaminophen exposure have focused on ASC and results are mixed [17, 32, 33]. We do not find evidence supporting this association in either ASC or ADHD symptoms but further research in larger samples is required.

The mechanisms proposed to underlie the adverse effects of early acetaminophen exposure on neurodevelopment include the stimulation of the endocannabinoid system, changes in brain-derived neurotrophic factor (BDNF) levels, oxidative stress due to inflammation-induced immune activation, changes in neurotransmission and endocrine-disruptive properties of acetaminophen [34, 35]. Acetaminophen exposure during periods equivalent to third trimester of pregnancy in humans but not later, induced behavioural and cognitive alterations in both male and female mice [36]. Other animal studies report findings that may be particularly interesting for ADHD. For instance, maternal exposure to acetaminophen was associated with lower levels of BDNF at the level of the striatum in an animal study conducted in male rats [37]. Furthermore, in male mice, acetaminophen treatment induced alterations in spatial learning, memory and dopamine metabolism [38]. Both the striatum region and dopamine are thought to play a pivotal role in ADHD [39–41].

The abovementioned findings provide biological plausibility and coherence for the current findings. In this regard, other causal criteria supported by the current findings include consistency and temporality [42]. Consistency is supported because we observed consistent results using a variety of populations and methods. Temporality is supported because the exposure precedes the onset of the symptoms assessed. Although we did not address dose-response relationship, previous studies have shown dose-response effects for both ASC and ADHD symptoms. [1, 6, 9]

Our findings need to be interpreted with caution given the limitations of our study. First, ASC and ADHD symptoms were assessed by different instruments in the cohorts. All instruments have been validated for the assessment of these symptoms, but the coverage may slightly differ among the instruments. To overcome this heterogeneity, we used instrument specific cut-offs to evaluate the presence or absence of borderline/clinical symptoms, a strategy that other meta-analysis analysing these outcomes have used [43, 44]. Although cohorts differed in the prevalence of ASC and ADHD symptoms, associations were largely consistent. Of note, despite all instruments used herein are widely used in the field but it would be important to examine the psychometric properties of the instruments used in each cohort to establish the specific objectivity of the outcomes. Second, confounding by indication cannot be completely ruled out although potential indications for acetaminophen use were included as covariates (maternal fever or infections during pregnancy, maternal chronic illnesses, and child cold or infections in the first 18 months of life). Third, dose and frequency of use were not harmonized across cohorts and therefore, not analysed herein. Fourth, although results were adjusted by several lifestyles and health factors that have been shown to be associated with prenatal acetaminophen exposure [45], residual confounding by social class cannot be completed discarded. However, the consistent associations found across different sensitivity analysis including examining ASC and ADHD diagnosis in the largest cohort makes unlikely that the observed relationship between prenatal acetaminophen and ASC and ADHD symptoms is entirely explained by unmeasured confounding. Fifth, given the high rates of loss to follow-up in some of the included cohorts, we cannot discard selection bias in some of the cohorts. Nevertheless, loss to follow-up was low (< 2%) in the largest cohort included when using hospital diagnosis, which had broadly consistent findings compared to all the other cohorts together. Sixth, we have exposure information on postnatal acetaminophen use up to different ages in different cohorts and one regional cohort only collected medication in case of infection, which may have caused some exposure misclassification in that cohort. Finally, although information was prospectively collected, we cannot rule out information bias or misclassification of exposure.

Overall, despite the above limitations, the homogeneity of the findings among the different cohorts, the novel assessment of postnatal acetaminophen exposure, and the use of an harmonized definition of exposure and outcome as well as of common statistical approaches overcomes the criticisms of previous meta-analysis [14, 15].

To conclude, our results support previous findings and address part of the weaknesses of previous meta-analyses. Considering all evidences on acetaminophen use and neurodevelopment, we agree with previous recommendations indicating that while acetaminophen should not be suppressed in pregnant women or children, it should be used only when necessary [46].

## Supplementary Information

Below is the link to the electronic supplementary material.

Supplementary file1 (DOCX 147 kb)

## Data Availability

Customized scripts in R software are available from the corresponding author upon request.

## Data availability

In order to protect participant's privacy, data at individual level cannot be made publicly available. For further details, authors can be contacted at http://www.isglobal.org/ca/.
