# Supplementary material for: Prenatal and postnatal exposure to acetaminophen in relation to autism spectrum and attention-deficit and hyperactivity symptoms in childhood: Meta-analysis in six European population-based cohorts
Source: Eur J Epidemiol. 2021 May 28;36(10):993–1004. doi: 10.1007/s10654-021-00754-4 (PMC8542535; doi:10.1007/s10654-021-00754-4)
Supplement: Supplementary file 1 — Supplementary file1 (DOCX 147 kb) [file 10654_2021_754_MOESM1_ESM.docx]

**SUPPLEMENTARY MATERIAL**

**Title:** Prenatal and postnatal exposure to acetaminophen in relation to autism spectrum and attention-deficit and hyperactivity symptoms in childhood: Meta-analysis in six European population-based cohorts

**Authors:** Silvia Alemany; Claudia Avella-García; Zeyan Liew; Raquel García-Esteban; Kosuke Inoue; Tim Cadman; Mònica López-Vicente; Llúcia González; Isolina Riaño Galán; Ainara Andiarena; Maribel Casas; Katerina Margetaki; Katrine Strandberg-Larsen; Deborah A. Lawlor; Hannan El-Marroun; Henning Tiemeier; Carmen Iñiguez; Adonina Tardón; Loreto Santa-Marina; Jordi Júlvez; Daniela Porta; Leda Chatzi; Jordi Sunyer.

**Table of contents**

Methods S1. Cohorts description. 3

Methods S2. Description of the acetaminophen prenatal exposure assessment 5

Methods S3. Description of the acetaminophen postnatal exposure assessment 7

Methods S4. Description of ASC symptoms assessment. 9

Methods S5. Description of ADHD symptoms assessment 11

Methods S6. Description of obtention of medical diagnosis of ASC and ADHD in DNBC cohort 13

Table S1. Psychometric properties of the instruments used to assess the outcomes of the study. 14

Table S2. Population characteristics by ASC status. 17

Table S3. Population characteristics by ADHD status. 18

Table S4. Population characteristics by prenatal acetaminophen exposure. 19

Table S5. Population characteristics by postnatal acetaminophen exposure. 20

Figure S1. Associations between early acetaminophen exposure and autistic autism spectrum condition (ASC) symptoms (A and C) and attention-deficit and hyperactivity (ADHD) symptoms (B and D) within the borderline/clinical range. Associations for prenatal (A and B) and postnatal (C and D) exposure are shown. 21

Table S6. Fully adjusted associations between early acetaminophen exposure and autistic spectrum (ASC) and attention-deficit and hyperactivity (ADHD) symptoms and hospital diagnosis for boys and girls. 22

Table S7. Sensitivity analysis examining the associations between early acetaminophen exposure and autistic spectrum (ASC) symptoms after excluding attention-deficit and hyperactivity (ADHD) cases. 23

Table S8. Sensitivity analysis showing associations between prenatal and postnatal acetaminophen exposure and autistic spectrum (ASC) symptoms and attention-deficit and hyperactivity (ADHD) symptoms adjusting for additional covariates. 24

Table S9. Leave-one-out sensitivity analyses: Adjusted associations between prenatal acetaminophen exposure and autistic spectrum (ASC) symptoms and attention-deficit and hyperactivity (ADHD) symptoms. 25

Table S10. Leave-one-out sensitivity analyses: Adjusted associations between postnatal acetaminophen exposure and autistic spectrum (ASC) symptoms and attention-deficit and hyperactivity (ADHD) symptoms. 26

# Methods S1. Cohorts description.

**ALSPAC**

The sample comprised participants from the Avon Longitudinal Study of Parents and Children (ALSPAC) (Boyd et al., 2013), an ongoing population-based study. The study website contains details of all data available through a fully searchable data dictionary and variable search tool (http://www.bristol.ac.uk/alspac/researchers/our-data/). Ethical approval for the study was obtained from the ALSPAC Ethics and Law Committee and the Local Research Ethics Committees. Informed consent for the use of data collected via questionnaires and clinics was obtained from participants following the recommendations of the ALSPAC Ethics and Law Committee at the time Pregnant women resident in Avon, UK with expected dates of delivery 1st April 1991 to 31st December 1992 were invited to take part in the study. The initial number of pregnancies enrolled is 14,541 (for these at least one questionnaire has been returned or a “Children in Focus” clinic had been attended by 19/07/99). Of these initial pregnancies, there was a total of 14,676 foetuses, resulting in 14,062 live births and 13,988 children who were alive at 1 year of age. For further details on the cohort profile, representativeness, and phases of recruitment, see (Boyd et al., 2013; Fraser et al., 2013; Northstone et al., 2019).

**DNBC**

The Danish National Birth Cohort (DNBC) is a nationwide longitudinal cohort that enrolled pregnant women through their general practitioners during early gestation (weeks 6 to 12) (Olsen et al., 2001). Written informed consent was obtained from all participants at recruitment, and again during followed up.

**GASPII**

The Gene and Environment: Prospective Study on Infancy in Italy (GASPII) is a national multi-center study conducted in Rome and Bologna (Porta & Fantini, 2006). Newborns were enrolled between June 2003 and October 2004 in Rome, and between June 2004 and December 2005 in Bologna. The study aims to test specific hypotheses on the aetiology of childhood diseases. Informed consent was obtained from all participants at enrollment and afterward at each follow-up. The study was approved by the Ethics Committee of the Università Cattolica del Sacro Cuore, Rome.

**The Generation R Study**

The Generation R Study is a population-based birth cohort study from fetal life onwards in Rotterdam, the Netherlands (Kooijman et al., 2016). The study is designed to identify early environmental and genetic causes and causal pathways leading to normal and abnormal growth, development and health from fetal life, childhood and young adulthood. The general design, all research aims and the specific measurements in the Generation R Study have been approved by the Medical Ethical Committee of Erasmus MC, University Medical Center Rotterdam. Participants need to give written informed consent for each phase of the study (fetal, preschool, childhood and adolescence period).

**INMA**

The INMA—INfancia y Medio Ambiente—(Environment and Childhood) Project is a network of birth cohorts from different Spanish regions that aim to study the role of environmental pollutants in air, water and diet during pregnancy and early childhood in relation to child growth and development (http://www.proyectoinma.org/) (Guxens et al., 2012). The study has been approved by Ethical Committee of each participating centre and written consent was obtained from participating parents. Data for this study comes from INMA-Asturias, INMA-Gipuzkoa, INMA-Sabadell and INMA-Valencia subcohorts.

**RHEA**

The Mother-Child Cohort in Crete (RHEA) is a prospective pregnancy cohort, at the prefecture of Heraklion, Crete, Greece (Chatzi et al., 2017). The study was approved by the Ethical Committee of the University Hospital of Heraklion (Crete, Greece), and all participants provided written informed consent after complete description of the study.

# Methods S2. Description of the acetaminophen prenatal exposure assessment

ALSPAC cohort

In ALSPAC, mothers received a questionnaire at 18 and 32 weeks of gestation including the following question: ‘Please indicate how often you have taken the following pills in the last three months’, (a) Aspirin, (b) Paracetamol, (c) Codeine, (d) Mogadon or other sleeping tablets, (e) Valium or other tranquilliser. Possible responses were as follows: everyday, most days, sometimes, not at all. For the present study we only considered paracetamol responses. Mothers were classified as users of acetaminophen during pregnancy if they had taken any dose of paracetamol at any time up to week 32 of pregnancy or the month before becoming pregnant. Further information can be found elsewhere (Golding et al., 2019).

DNBC cohort

In DNBC, information about maternal acetaminophen use during pregnancy was ascertained from the study enrolment form and three computer-assisted telephone interviews. At the first contact, women filled out a form that included questions regarding any supplement and medication use covering the period from 4 weeks before pregnancy to the gestational week of reporting. In the subsequent telephone interviews (scheduled around 12^th^ and 30^th^ week, and 6-month postpartum), women were specifically asked to report whether they had taken any pain killers during pregnancy provided with a list of 44 common medications, including acetaminophen as a single or combination drug (Liew, Ritz, Rebordosa, Lee, & Olsen, 2014). Mothers were classified as users of acetaminophen during pregnancy if they had taken any dose of acetaminophen at any time up during pregnancy. Otherwise, they were considered non-exposed.

GASPII cohort

In GASPII cohort, information about maternal acetaminophen use at each trimester of the pregnancy was retrospectively assessed in interviews conducted at birth. Mothers were classified as users of acetaminophen during pregnancy if they had taken any dose of acetaminophen at any time up to week 32 of pregnancy or the month before becoming pregnant. Otherwise, they were considered non-exposed.

Generation R cohort

In Generation R cohort, three self-administered questionnaires assessing medication use during pregnancy were sent out by post at gestational weeks, 12, 20 and 30 (the average lag period to response was 2 weeks). The first questionnaire contained the following question ‘Did you use any medication during the past 6 months?’. This question was followed by a scheme in which mothers had to fill out the name of the medication, reason for use, age at the start of use, use during pregnancy, and if they stopped use when the pregnancy was known. The second questionnaire contained the following question ‘Did you use any medication during the past 2 months?’ and the third questionnaire contained a similar question but focusing on the past 3 months. Mothers were classified as users of acetaminophen during pregnancy if they had taken any dose of acetaminophen at any time up to week 32 of pregnancy or the month before becoming pregnant. Otherwise, they were considered non-exposed. Further information can be found elsewhere (Snijder et al., 2012).

INMA cohort

In INMA cohort, mothers were interviewed face-to-face at weeks 12 and 32 of pregnancy using standardized questionnaires completed by trained evaluators. At each interview mothers were asked if they had taken medications. Specifically, at week 12 mothers were asked ‘Have you taken any medication (sporadically or continuously) since 1 month before becoming pregnant or during this pregnancy?’. Similarly, at week 32, mothers were asked ‘Have you taken any medication (sporadically or continuously) since the last interview?’ For both questions, if the answer was positive, the name of the medication, dose, duration, gestational age at use and the indication as reported by the mother were enquired using open questions. Data were coded using the Pharmacological Vade Mecum officially recognized by the Spanish Ministry of Health. Acetaminophen could have been used as a single drug or as a fixed-dose combination. Mothers were classified as users of acetaminophen during pregnancy if they had taken any dose of acetaminophen at any time up to week 32 of pregnancy or the month before becoming pregnant. Otherwise, they were considered non-exposed. Further information can be found elsewhere (Avella-Garcia et al., 2016).

RHEA cohort

In RHEA cohort, information on medication intake was obtained via face-to-face interviews at weeks 12 and 30 of pregnancy. Specifically, at week 12 mothers were asked ‘Except any medical treatment you maybe following for specific chronic diseases, have you ever taken also any other medication, from the moment you got pregnant until now?’. Similarly, at week 30, mothers were asked ‘Apart from the medicine that you take for chronic illnesses, have you taken any other medicine (for example analgetics, antipyretics, counterirritants, antibiotics) during the last 3 months?’. In all three questions the mothers were asked to report the drug name, the cause/disease, the start date and the end date, the frequency, the dosage and who suggested it. From this information a binary variable indicating whether the mother was exposed or not to acetaminophen exposure during pregnancy was derived.

# Methods S3. Description of the acetaminophen postnatal exposure assessment

ALSPAC cohort

In ALSPAC, information of medication administered to the children was assessed when children were 6, 15 and 24 months old through maternal reports. Children were classified as exposed to postnatal acetaminophen if mothers indicated administration of any dose of acetaminophen at any time up to 24 months of life. Otherwise, they were considered non-exposed.

DNBC cohort

In DNBC, information of medication administered to the children was assessed through computer-assisted telephone interviews with mothers when children were 6 and 18 months old. Mothers were asked to report whether their child had experienced any of 16 types of conditions or diseases and whether and the specific pharmaceutical treatment for these conditions (Fever >38.5, colds, ear infection, wheezy breathing, throat inflammation, false croup, colic, pneumonia, eczema or skin eruptions, bronchitis, diarrhoea, constipation, scarlet fever, eye inflammation, fungus in mouth, pruritic skin eruption, other and unspecified). Children were classified as exposed to postnatal acetaminophen if mothers indicated administration of any dose of acetaminophen at any time up to 18 months of life. Otherwise, they were considered non-exposed.

GENERATION R cohort

In Generation R cohort, medication administered to the children was reported twice through questionnaires. The first questionnaire, completed when children were 6 months of age, contained the following question ‘Has your child ever used one or more of the following medicines?’. In the second questionnaire that was completed at 12 months child age, the question was ‘Has your child used one or more of the following medicines in the last 6 months?’. Children were classified as exposed to postnatal acetaminophen if mothers indicated administration of any dose of acetaminophen at any time up to 12 months of life. Otherwise, they were considered non-exposed.

GASPII cohort

In GASPII cohort, mothers were asked if any medication had been administered to their children at 6 and 15 months of life. The first questionnaire included the following question: “In the first 6 months of life, did your child need to take paracetamol?”. In the second questionnaire, the question was “Between the 6th and 15th month of life, did your child need to take paracetamol?”. Children were classified as exposed to postnatal acetaminophen if mothers indicated administration of any dose of acetaminophen at any time up to 15 months of life. Otherwise, they were considered non-exposed.

INMA cohort

In INMA cohort, mothers were asked to indicate medications administered to their children through questionnaires. Periods of exposures varied across INMA subcohorts. In INMA-Sabadell and INMA-Asturias, mothers indicated whether their children were administered any medication in the first 6 months of life through self-reported questionnaires. In INMA-Sabadell, mothers reported if any medication was administered to children from 6 to 14 months of life. In INMA-Asturias, mothers reported if children were administered any medication for infections from 6 to 18 months of life. In INMA-Gipuzkoa, mothers reported if children were administered any medication from birth to 14 months. Thus, the overlapping period for which medication use (any medication or medication for infections or bronchiolitis, bronchitis or pneumonia) was available for the children across INMA subcohorts extends from birth to 14 months of life. Children were classified as exposed to postnatal acetaminophen if mothers indicated administration of any dose of acetaminophen at any time up to 14 months of life. Otherwise, they were considered non-exposed. Postnatal acetaminophen use was not collected in Valencia subcohort.

RHEA cohort

In RHEA cohort, mothers reported if any medication had been administered to their children at 9 months of life via face-to-face interviews. The specific question they answered was ‘Apart from the medicine that your child took for the above illnesses, have they taken any other medicine (for example analgetics, antipyretics, counterirritants, antibiotics)?’. Children were classified as exposed to postnatal acetaminophen if mothers indicated administration of any dose of acetaminophen at any time up to 9 months of life. Otherwise, they were considered non-exposed.

# Methods S4. Description of ASC symptoms assessment.

Social Communication Conditions Checklist (SCDC) (ALSPAC)

In ALSPAC, autistic traits were assessed using the validated parent-reported Social Communication Conditions Checklist (SCDC; (D H Skuse, Mandy, & Scourfield, 2005)). This instrument consists of 13 items each describing a specific behaviour. Parents need to indicate whether the statement properly describes the behaviour of the child in the past 6 months in a three-point Likert scale (0, not true; 1, quite or sometimes true; 2, very or often true). Higher scores indicate more autistic traits. It has high reliability and internal consistency and good discriminant validity between pervasive developmental disorder and other clinical groups. Discriminant validity, measured in a clinical population, predicted autism with a sensitivity of 0.88 and specificity of 0.91 when a cut-off of 8 or above is used to detect probable cases (David H. Skuse et al., 2009).

Strengths and Difficulties Questionnaire (SDQ) (DNBC)

In DNBC, autistic traits were assessed using the Strengths and Difficulties Questionnaire (SDQ, (Robert Goodman, 1997)) when children were 7 years old. The SDQ is a brief behavioural screening of 25 items for individuals aged between 4 and 16 years old. Main caregivers were asked to indicate on a 3-point response scale (ranging from not true to certainly true) how well each item described their child behaviour during the previous 6 months. The questionnaire consists of five subscales including emotional problems, peer problems, conduct problems, hyperactivity and prosocial behaviour, each of them composed by 5 items. Each subscale score is computed by summing up its 5 corresponding items. A supplemental subscale referred as ‘impact’ measures chronicity, distress, social impairment, and burden to others. Higher scores indicate more behavioural problems except for prosocial behaviour subscale. The predictive value of the combination of different SDQ subscales for clinical diagnosis of ASC has been investigated (Russell, Rodgers, & Ford, 2013). Abnormal scores in emotional problems and in peer problems or prosocial behaviour SDQ subscales were recommended to capture ASC symptoms (Russell et al., 2013).

Pervasive Developmental Problems (PDP) subscale of the Child Behavior Checklist for

Toddlers (CBCL1½-5) (GASP and Generation R)

In GASP and Generation R Study cohorts, the CBCL1½-5 was used to assess behaviour and emotional problems during early childhood (Thomas M . Achenbach, 2000). The Dutch and the Italian versions are reliable and validated (Muratori et al., 2011; Tick, Van der Ende, Koot, & Verhulst, 2007). The subscales for syndromes derived from the CBCL1½-5 are consistent with diagnostic categories of the Diagnostic and Statistical Manual of Mental Disorders, 4th edition (DSM-IV; American Psychiatric Association, 2002). The pervasive developmental problems (PDP) subscale is a DSM-oriented scale consisting of 13 items aimed to identify children at risk for autism spectrum conditions. Each item of the questionnaire describes a specific behaviour and the parent is asked to rate its frequency on a three-point Likert scale (0, not true, 1, somewhat or sometimes true, 2, very true or often true). Higher scores indicate more autistic traits. The PDP subscale has a good predictive validity to identify children at risk of autism spectrum conditions (Sikora et al. 2008), with areas under the receiving operating characteristic (ROC) curve of 0.95 (Muratori et al., 2011). We used the 93rd percentile as cut-off scores to classify children with autistic traits within the borderline/clinical range (Tick et al., 2007).

Childhood Autism Spectrum Test (CAST) (INMA cohort)

In INMA cohort, the Childhood Autism Spectrum Test (CAST) was administered to the parents by a psychologist to assess autistic traits of the child. This questionnaire is based on behavioural descriptions of the ICD-10 (WHO 1993) and Diagnostic and Statistical Manual of Mental Disorders, 4th edition (DSM-IV) criteria (APA 2000) designed to identify subtle manifestations of autism spectrum conditions (social impairments, communication impairments and repetitive or stereotyped behaviours) in the mainstream school population (Scott, Baron-Cohen, Bolton, & Brayne, 2002; Williams et al., 2005). The questionnaire includes 37 key items coded as ‘No’ or ‘Yes’ that contribute to a total score, along with 6 control questions on general development. Higher scores indicate more autistic traits. The CAST has shown high sensitivity among schoolchildren (Williams et al., 2005). We applied cut-offs to yield proxies for autistic traits within the borderline or clinical range at 12 points that correspond to a sensitivity 0.89 and specificity of 0.80 (Morales-Hidalgo, Roigé-Castellví, Vigil-Colet, & Canals Sans, 2017).

Autism spectrum disorders Scale from the Child Behaviour Checklist for Toddlers (ASC-CBCL6-18 scale) (RHEA)

In RHEA cohort, no specific instrument assessing ASC traits was available. For this reason, we assessed ASC symptoms using a recently proposed combination of 9 items from the CBCL 6-18 to generate an ASC-CBCL scale including: acts too young for his/her age (item 1), doesn’t get along with other kids (item 25), fears certain animals, situations (item 29), would rather be alone than with others (item 42), nervous movements or twitching (item 46), repeats certain acts over and over, compulsions (item 66), speech problem (item 79), strange behaviour (item 84, and withdrawn, doesn’t get involved with others (item 111) (Ooi, Rescorla, Ang, Woo, & Fung, 2011). The ability of this scale to discriminate children diagnosed with ASC was assessed in an Asian sample consisting of 86 children with ASC, 117 children with ADHD-Inattentive type, 426 children with ADHD-Hyperactive-impulsive or combined type, 200 clinically referred children who did not receive diagnosis and 436 typically developing children. Sensitivity for this 9-item ASC scale ranges from 68 to 78% and specificity ranges from 73 to 92%. Up to our knowledge, there is no validated cut-off for this specific scale. We applied the recommended cut-offs for the CBCL to classify children within borderline/clinical range corresponding to scores above 93^rd^ percentile (T.M Achenbach & Rescorla, 2001). Although this cut-off corresponds to T-scores for the standardized scales of the CBCL, when applied in RHEA, 9.94% of the children were classified as borderline clinical cases of ASC. This figure was similar to the prevalence found in GASP (12.7%) and INMA (6.75).

# Methods S5. Description of ADHD symptoms assessment

DAWBA (ALSPAC)

In ALSPAC cohort, ADHD symptoms were assessed using the Development and Well-Being Assessment (DAWBA) (R Goodman, Ford, Richards, Gatward, & Meltzer, 2000), a semi-structured interview including child and parent interviews alongside a teacher questionnaire. The child/parent interviews and teacher questionnaires assess current and recent past psychiatric symptoms and their impact on functioning in children. The DAWBA is based on diagnostic criteria (ICD-10 and DSM-IV) and focuses on anxiety disorders, depressive disorders, ADHD and conduct disorders. A clinical diagnostic rating is informed by triangulation of these three sources. The validity of the clinical diagnoses derived from the DAWBA have been shown by concordance with case note screening in a clinical sample of children aged 11–15 years, and with a full clinical assessment for ADHD specifically (Foreman, Morton, & Ford, 2009).

SDQ (DNBC cohort)

In DNBC, ADHD traits were assessed using the Strengths and Difficulties Questionnaire (SDQ, (Robert Goodman, 1997)) when children were 7 years old. The SDQ is a brief behavioural screening of 25 items for individuals aged between 4 and 16 years old. Main caregivers were asked to indicate on a 3-point response scale (ranging from not true to certainly true) how well each item described their child behaviour during the previous 6 months. The questionnaire consists of five subscales including emotional problems, peer problems, conduct problems, hyperactivity and prosocial behaviour, each of them composed by 5 items. Each subscale score is computed by summing up its 5 corresponding items. A supplemental subscale referred as ‘impact’ measures chronicity, distress, social impairment, and burden to others. Higher scores indicate more behavioural problems except for prosocial behaviour subscale. The predictive value of the combination of different SDQ subscales for clinical diagnosis of ADHD has been investigated (Russell et al., 2013). Abnormal scores in hyperactivity/inattention and impact subscales were recommended to capture ADHD (Russell et al., 2013).

DSM-oriented attention deficit/hyperactivity problems subscale of the Child Behavior

Checklist for Toddlers (CBCL1½-5) (GASP cohort)

In GASP and Generation R Study cohorts, the CBCL1½-5 was used to assess behaviour and emotional problems during early childhood (Thomas M . Achenbach, 2000). Reliability and validity of the Italian version have been established (Muratori et al., 2011). The subscales for syndromes derived from the CBCL1½-5 are consistent with diagnostic categories of the Diagnostic and Statistical Manual of Mental Disorders, 4th edition (DSM-IV; American Psychiatric Association, 2002). The attention deficit/hyperactivity problems subscale is a DSM-oriented scale consisting of 6 items aimed to identify children at risk for ADHD. Each item of the questionnaire describes a specific behaviour and the parent is asked to rate its frequency on a three-point Likert scale (0, not true, 1, somewhat or sometimes true, 2, very true or often true). Higher scores indicate more ADHD symptoms. This subscale is a useful screening instrument to identify children with ADHD (Tick et al., 2007). We used the 93rd (>8 points) percentile as cut-off scores to classify children with ADHD symptoms within the borderline/clinical range (Tick et al., 2007).

Conner’s Rating Scale Revised short form (CPRS-R:S) (Generation R cohort)

In Generation R cohort, ADHD symptoms were assessed at age 8 using the ADHD index from the short form of the Conners' Parent Rating Scales (CSRS-R:S,(Conners, 1997)). The CPRS-R:S consists of 27 items rated on a 4-point Likert scale (ranging from 0=not true at all to 3=very much true) intended to assess problematic behaviour over the last month in children aged 6-18 years. The CPRS-R:S provides three subscales (oppositional, cognitive problems/inattention and hyperactivity) and an ADHD index. The ADHD Index is a 10-item scale that assesses the presence of the most prominent symptoms of ADHD. The crude scores of this test can be translated into T-scores, which are age and gender specific (American normative sample). The scale has a cut-off for elevated scores (T 65-69) that we used to classify children as borderline/clinical cases (T-scores >65) or controls (T-scores <65) (Conners, 1997).

DSM-ADHD Questionnaire (INMA cohorts)

In INMA cohort, ADHD symptoms were assessed using a questionnaire based on the DSM-IV-ADHD criteria (DSM-IV; American Psychiatric Association, 2002) at age 4 reported by teachers (INMA). The ADHD-DSM-IV questionnaire consists of a list of 18 symptoms, assessing two separate symptom groups: inattention (nine symptoms) and hyperactivity/impulsivity (nine symptoms). Each ADHD symptom is rated on a 4-point frequency scale from never or rarely (0) to very often (3). The ADHD symptoms score ranges from 0 to 54, with higher scores indicating more symptoms. We applied the established cut-offs to yield proxies for ADHD symptoms within the clinical range (≥6 symptoms of inattention or hyperactivity) (American Psychiatric Association, 2002).

ADHD DSM-oriented scale of the Child Behaviour Checklist for Toddlers (CBCL 6-18) (RHEA cohort)

In RHEA cohort, ADHD symptoms were assessed at age 6 using the ADHD DSM-oriented scale from the CBCL 6/18 (T.M Achenbach & Rescorla, 2001) reported by parents. The CBCL 6-18 is widely used standardized parent report questionnaire consisting of 113 items. Each item is scored on a 3-point Likert scale (0 = Not True, 1 = Somewhat or Sometimes True, or 2 = Very True or Often True) to describe=the child's behavior during the preceding 6 months. All CBCL scales have a T-score mean of 50 and standard deviation of 10 and different norms are provided for each gender across the two normative age groups 6–11 and 12–18 years. The

CBCL provides eight syndrome scales (anxious/depressed, withdrawn/depressed, somatic complaints, social problems, thought problems, attention problems, rule-breaking behavior, and aggressive behavior) and DSM-Oriented scores (depressive problems, anxiety problems, somatic problems, attention deficit, oppositional defiant problems and conduct problems).

It has been reported that the DSM-oriented outperforms the attention problems syndrome scale as screening instrument for ADHD in community and clinical samples (Aebi, Winkler Metzke, & Steinhausen, 2010). Children with CBCL/6–18 T-scores above 65 on ADHD DSM-oriented scale were classified as borderline/clinical cases, children with scores on this scale below 65 were classified as controls (T.M Achenbach & Rescorla, 2001).

# Methods S6. Description of obtention of medical diagnosis of ASC and ADHD in DNBC cohort

Hospital diagnosis of ASC and ADHD were obtained by linking DNBC to the Danish National Hospital Registry (Andersen, Madsen, Jørgensen, Mellemkjær, & Olsen, 1999) which contains nationwide records for all somatic admissions, and also the Danish Psychiatric Central Registry (Munk‐Jørgensen, Kastrup, & Mortensen, 1993) for all inpatient and outpatient admissions to psychiatric hospitals. The linkage was performed on 31^st^ October 2013 with an average of 12.8 years of follow up (Liew, Ritz, Virk, & Olsen, 2016). All diagnoses are based on the International Classification of Diseases 10th Edition (ICD-10 F84.0-F84.9 for Autism Spectrum Condition and ICD10 F90.0-F90.9 for hyperkinetic disorders. Hyperkinetic Disorder defined in the ICD-10 is comparable to the severe form of Attention-deficit/hyperactivity disorder diagnosis according to Diagnostic and Statistical Manual-IV (Lee et al., 2008).

# Table S1. Psychometric properties of the instruments used to assess the outcomes of the study.

| **Instrument** | **Cohorts using the instrument** | **Outcome** | **Validity and reliability** | **Internal consistency** | **Test re-test reliability** | **Discriminant validity** | **References** |
| --- | --- | --- | --- | --- | --- | --- | --- |
| Social Communication Conditions Checklist (SCDC) | ALSPAC | ASC | Tamhane’s T2 test showed significant differences in SCDC scores between three clinical groups (community controls, clinical controls and ASD cases) (P<0.001) | Cronbach’s coefficient= 0.93 | The ICC for test–retest SCDC scores on a clinical sample of 188 children with a mean retest interval of 2.7 years was 0.81 (95% CI 0.76–0.86). | The sensitivy and specifivity for the cut-off of 8 points were 0.88 and 0.91, respectively. | (Skuse, Mandy, & Scourfield, 2005) |
| Childhood Autism Spectrum Test (CAST) | INMA | ASC | Positive correlations with ADI-R (0.42-0.57) and total score of ADOS-2 (0.41). | The Cronbach’s alpha for CAST total score was 0.64 (good to moderate). | Kappa of 0.82 for a test-retest across three score groups (≥ 15; 12–14; <12) with an interval of 3 weeks. | The sensitivity and specificity for the cut-off of 12 points was 0.89 and 0.80, respectivelys | (Morales-Hidalgo, Roigé-Castellví, Vigil-Colet, & Canals Sans, 2017; Williams et al., 2005) |
| Strengths and Difficulties Questionnaire (SDQ) | DNBC | ASC and ADHD | Positive correlations were found between SDQ subscales and Rutter questionnaire (0.78 to 0.88). High SDQ scores (in the extreme 10% of the population) were associated with increased psychiatric risk (OR=15) | Average Cronbach’s coefficient for all subscale = 0.73 | Mean retest stability of 0.62 after 4 to 6 months | Using a combination of abnormal scores from the subscales, the model for ASC showed a sensitivity of 0.79 and specificity of 0.93. The model for ADHD showed a sensitivity of 0.91 and specificity of 0.90. | (R Goodman, 2001; Robert Goodman, 1997; Russell, Rodgers, & Ford, 2013) |
| Conner’s Rating Scale Revised short form (CPRS-R:S) | Generation R Study | ADHD | Correlations with CBCL 6-18-ADHD-DSM scale,externalizing and total problems were between .60 and .76 (Spanish version). | Internal reliability: 0.90 | - | Recommended cut-offs for elevates scores (T=65-69) | (Conners, 2008; Morales-Hidalgo, Hernández-Martínez, Vera, Voltas, & Canals, 2017) |
| DSM-ADHD Questionnaire | INMA | ADHD | - | Cronbach’s alpha coefficient= 0.90 | - | - | (American Psychiatric Association., 2002; Ramos et al., 2013) |
| Child Behaviour Checklist for Toddlers (CBCL 6-18) | GASPII and Generation R Study | ASC and ADHD | Significant associations with analogous scales of other instruments and with DSM criteria. | Cronbach's alpha for ADHD-DSM oriented scale= 0.84. Cronbach's alpha for ASD scale=0.64. | Mean test re-test correlation of 0.93 with intervals of 8 to 16 days. | Recommended cut-off 93th percentile for borderline-clinical symptoms | (T. . Achenbach & Rescorla, 2001; Ooi, Rescorla, Ang, Woo, & Fung, 2011) |
| Child Behavior Checklist for Toddlers (CBCL1½-5) | RHEA | ASC and ADHD | Significant associations with analogous scales of other instruments and with DSM criteria. | Cronbach's alpha for ADHD-DSM oriented scale= 0.79 in a Spanish sample. | Mean test re-retest correlation=0.85 with intervals of 8 days. | Recommended cut-off 93th percentile for borderline-clinical symptoms. The sensitivity and specificity for PDP were 0.85 and 0.90, respectively, in an Italian sample. | (T. Achenbach & Rescorla, 2000; de la Osa, Granero, Trepat, Domenech, & Ezpeleta, 2016; Muratori et al., 2011) |
| Development and Well-Being Assessment (DAWBA) | ALSPAC | ADHD | DAWBA diagnosed the same disorder in 93% of the cases. The prevalence estimates of the computer-generated DAWBA diagnoses were of roughly comparable magnitude to the prevalence estimates from the clinician-generated diagnoses | - | - | Sensitivity and specificity of 0.80 for DAWBA diagnosis of DSM-ADHD. | (Foreman, Morton, & Ford, 2009; A. Goodman, Heiervang, Collishaw, & Goodman, 2011; R Goodman, Ford, Richards, Gatward, & Meltzer, 2000) |
| NOTE: ADHD, attention-deficit and hyperactivity disorder; ASC, autism spectrum conditions; ADI-R, Autism Diagnostic Interview Revised; ADOS-2, Autism Diagnostic Observation Schedule-Second Edition; DSM, Diagnostic and Statistical Manual of Mental Disorders; PDP, pervasive developmental disorder. | | | | | | | |

# Table S2. Population characteristics by ASC status.

# Table S3. Population characteristics by ADHD status.

# Table S4. Population characteristics by prenatal acetaminophen exposure.

# Table S5. Population characteristics by postnatal acetaminophen exposure.

#
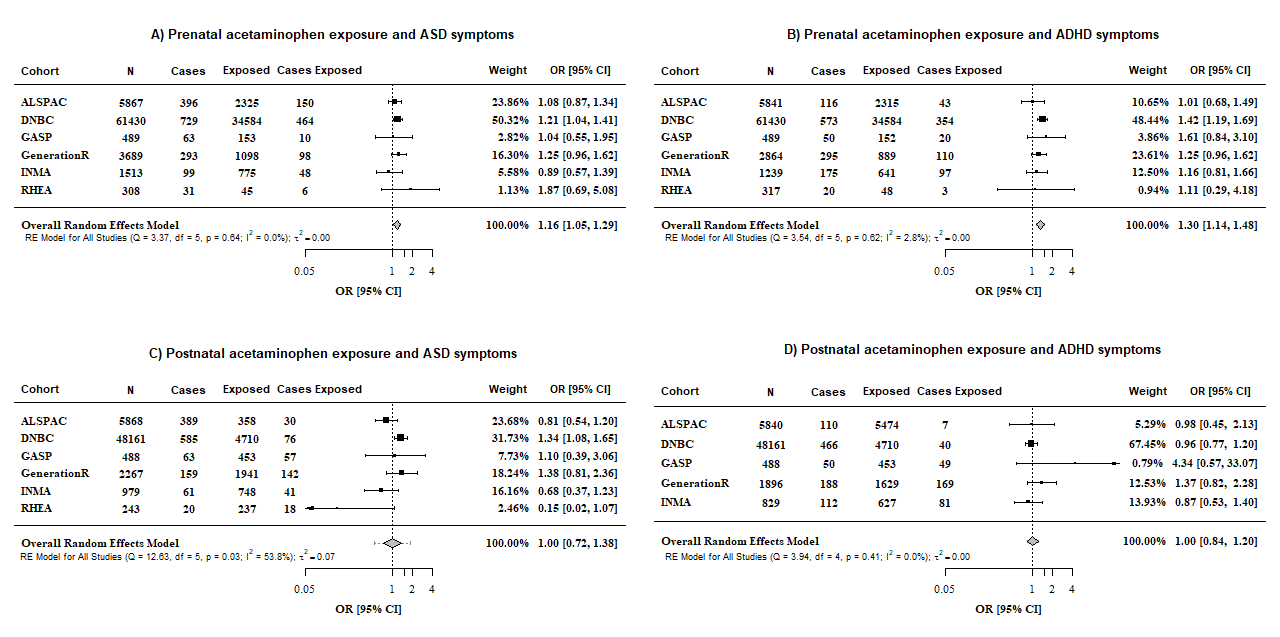
Figure S1. Associations between early acetaminophen exposure and autistic autism spectrum condition (ASC) symptoms (A and C) and attention-deficit and hyperactivity (ADHD) symptoms (B and D) within the borderline/clinical range. Associations for prenatal (A and B) and postnatal (C and D) exposure are shown.

NOTE: Analysis in DNBC used hospital diagnosis of ASC and ADHD as outcomes. Odds Ratio (OR) and 95% confidence intervals (CI) by cohort and overall estimate obtained from random-effects meta-analysis. Models were adjusted for maternal characteristics (education, age at delivery, pre-pregnancy body mass index, prenatal smoking, mental health during pregnancy, parity and alcohol consumption, fever and infections during pregnancy) and child’s characteristics (sex, age at the behavioural assessment). Postnatal models were further adjusted by child’s cold and respiratory infections. Models on postnatal exposure and ADHD symptoms were not possible to conduct in RHEA cohort (limited sample size).

| Table S6. Fully adjusted associations between early acetaminophen exposure and autistic spectrum (ASC) and attention-deficit and hyperactivity (ADHD) symptoms and hospital diagnosis for boys and girls. | | | | | | | | | | | | | | |
| --- | --- | --- | --- | --- | --- | --- | --- | --- | --- | --- | --- | --- | --- | --- |
|  |  |  | **BOYS** | | | | |  |  | **GIRLS** | | | | |
| **Exposure period** | **Outcome** | **n^a^** | **N^b^** | **OR (95 % CI)^c^** | ***I^2^* (%)** | **Q-value** | **P-heter** |  | **n^a^** | **n^b^** | **OR (95 % CI)^c^** | ***I^2^* (%)** | **Q-value** | **P-heter** |
| **Prenatal** | **ASC symptoms and diagnosis** ^d^ | 6 | 30614 | 1.14 (1.00, 1.29) | 0.01 | 4.488 | 0.482 |  | 5 | 29516 | 1.15 (0.78, 1.71) | 68.37 | 11.737 | 0.019 |
|  | **ADHD symptoms and diagnosis** ^d^ | 4 | 30032 | 1.31 (1.15, 1.49) | 0.00 | 2.730 | 0.742 |  | 5 | 28975 | 1.39 (1.15, 1.68) | 0.00 | 1.703 | 0.790 |
| **Postnatal** | **ASC symptoms and diagnosis** ^d^ | 6 | 29481 | 1.16 (0.88, 1.53) | 20.87 | 13.874 | 0.016 |  | 5 | 28520 | 0.86 (0.55, 1.34) | 34.47 | 6.037 | 0.196 |
|  | **ADHD symptoms and diagnosis** ^d^ | 4 | 30032 | 1.12 (0.91, 1.38) | 0.01 | 4.221 | 0.239 |  | 5 | 28172 | 0.71 (0.50, 1.03) | 0.00 | 2.364 | 0.669 |
| NOTE:  *I^2^*, percentage of the total variability due to between-study heterogeneity; P-Heter, P-value of heterogeneity using the Cochran’s Q test.  ^a^Number of cohorts included in the meta-analysis. ^b^Number of children included in the meta-analysis.  ^c^Odds ratios and 95% confidence intervals were estimated by random-effects meta-analysis. Models were adjusted for maternal characteristics (education, age at delivery, pre-pregnancy body mass index, prenatal smoking, mental health during pregnancy, parity and alcohol consumption, fever and infections during pregnancy) and child’s characteristics (sex, age at the behavioral assessment). Postnatal models were further adjusted by child’s cold and respiratory infections.  ^d^ASC and ADHD symptoms within the borderline/clinical range were assessed using parental and teacher reported questionnaires in all cohorts except DNBC, where hospital diagnosis were also available. | | | | | | | | | | | | | | |

# Table S7. Sensitivity analysis examining the associations between early acetaminophen exposure and autistic spectrum (ASC) symptoms after excluding attention-deficit and hyperactivity (ADHD) cases.

|  | | | | | | | |
| --- | --- | --- | --- | --- | --- | --- | --- |
| **Exposure period** | **Outcome** | **n^a^** | **N^b^** | **OR (95 % CI)^c^** | ***I^2^* (%)** | **Q-value** | **P-heter** |
| **Prenatal** | **ASC symptoms^d^** | 6 | 71897 | 1.16 (1.01, 1.43) | 0.00 | 4.918 | 0.426 |
|  | **ASC symptoms and diagnosis^e^** | 6 | 58494 | 1.09 (0.97, 1.23) | 0.00 | 4.583 | 0.469 |
| **Postnatal** | **ASC symptoms^d^** | 6 | 56967 | 0.98 (0.69, 1.40) | 51.09 | 12.456 | 0.029 |
|  | **ASC symptoms and diagnosis^e^** | 6 | 56505 | 0.98 (0.70, 1.37) | 47.42 | 11.762 | 0.038 |
| NOTE:  *I^2^*, percentage of the total variability due to between-study heterogeneity; P-Heter, P-value of heterogeneity using the Cochran’s Q test.  ^a^Number of cohorts included in the meta-analysis. ^b^Number of children included in the meta-analysis.  ^c^Odds ratios and 95% confidence intervals were estimated by random-effects meta-analysis. Models were adjusted for maternal characteristics (education, age at delivery, pre-pregnancy body mass index, prenatal smoking, mental health during pregnancy, parity and alcohol consumption, fever and infections during pregnancy) and child’s characteristics (sex, age at the behavioral assessment). Postnatal models were further adjusted by child’s cold and respiratory infections.  ^d^ASC and ADHD symptoms within the borderline/clinical range were assessed using parental and teacher reported questionnaires in all cohorts.  ^e^ASC and ADHD symptoms within the borderline/clinical range were assessed using parental and teacher reported questionnaires except DNBC, where medical diagnosis were also available. | | | | | | | |

# Table S8. Sensitivity analysis showing associations between prenatal and postnatal acetaminophen exposure and autistic spectrum (ASC) symptoms and attention-deficit and hyperactivity (ADHD) symptoms adjusting for additional covariates.

| **Exposure period** | **Outcome** | **n^a^** | **N^b^** | **OR (95 % CI)^c^** | ***I^2^* (%)** | **Q-value** | **P-heter** |
| --- | --- | --- | --- | --- | --- | --- | --- |
| **Prenatal** | **ASC symptoms^d^** | 6 | 72370 | 1.21 (1.08, 1.35) | 0.01 | 4.385 | 0.496 |
|  | **ASC symptoms and diagnosis^e^** | 6 | 59489 | 1.18 (1.06, 1.31) | 0.02 | 3.845 | 0.572 |
|  | **ADHD symptoms^d^** | 5 | 70988 | 1.22 (1.07, 1.38) | 0.00 | 1.692 | 0.792 |
|  | **ADHD symptoms and diagnosis^e^** | 5 | 58107 | 1.33 (1.17, 1.51) | 0.00 | 2.666 | 0.615 |
| **Postnatal** | **ASC symptoms^d^** | 6 | 57287 | 0.99 (0.72, 1.37) | 50.55 | 12.164 | 0.033 |
|  | **ASC symptoms and diagnosis^e^** | 6 | 57801 | 0.99 (0.72, 1.37) | 53.5 | 12.905 | 0.024 |
|  | **ADHD symptoms^d^** | 5 | 56533 | 0.95 (0.75, 1.21) | 4.69 | 4.922 | 0.682 |
|  | **ADHD symptoms and diagnosis^e^** | 5 | 56533 | 1.00 (0.83, 1.20) | 0.00 | 3.999 | 0.406 |
| NOTE:  *I^2^*, percentage of the total variability due to between-study heterogeneity; P-Heter, P-value of heterogeneity using the Cochran’s Q test.  ^a^Number of cohorts included in the meta-analysis. ^b^Number of children included in the meta-analysis.  ^c^Odds ratios and 95% confidence intervals were estimated by random-effects meta-analysis. Models were adjusted for maternal characteristics (education, age at delivery, pre-pregnancy body mass index, prenatal smoking, mental health during pregnancy, parity and alcohol consumption, fever and infections during pregnancy, chronic diseases, use of other drugs and use of folic acid) and child’s characteristics (sex, age at the behavioral assessment, birthweight, gestational age). Postnatal models were further adjusted by child’s cold and respiratory infections.  ^d^ASC and ADHD symptoms within the borderline/clinical range were assessed using parental and teacher reported questionnaires in all cohorts.  ^e^ASC and ADHD symptoms within the borderline/clinical range were assessed using parental and teacher reported questionnaires except DNBC, where hospital diagnosis were also available. | | | | | | | |

# Table S9. Leave-one-out sensitivity analyses: Adjusted associations between prenatal acetaminophen exposure and autistic spectrum (ASC) symptoms and attention-deficit and hyperactivity (ADHD) symptoms.

|  | **ASC symptoms^a^** | | | |  | **ADHD symptoms^a^** | | | |
| --- | --- | --- | --- | --- | --- | --- | --- | --- | --- |
| **Cohort omitted** | **OR (95 % CI)^b^** | ***I^2^* (%)** | **Q-value** | **P-heter** |  | **OR (95 % CI)^b^** | ***I^2^* (%)** | **Q-value** | **P-heter** |
| **ALSPAC** | **1.24 (1.09, 1.4)** | 0,00 | 3.21 | 0.52 |  | **1.23 (1.08, 1.4)** | 0,00 | 0.8 | 0.94 |
| **DNBC** | 1.12 (0.96, 1.3) | 0,00 | 2.85 | 0.58 |  | **1.19 (1, 1.42)** | 0,00 | 1.7 | 0.79 |
| **GASP** | **1.2 (1.07, 1.34)** | 0,00 | 4.14 | 0.39 |  | **1.19 (1.05, 1.35)** | 0,00 | 0.95 | 0.92 |
| **GenerationR** | **1.16 (1, 1.35)** | 0.01 | 4.21 | 0.38 |  | **1.19 (1.04, 1.37)** | 0,00 | 1.63 | 0.8 |
| **INMA** | **1.22 (1.09, 1.36)** | 0,00 | 2.58 | 0.63 |  | **1.21 (1.06, 1.38)** | 0,00 | 1.67 | 0.8 |
| **RHEA** | **1.18 (1.05, 1.33)** | 0,00 | 3.55 | 0.47 |  | **1.21 (1.07, 1.37)** | 0,00 | 1.71 | 0.79 |
| NOTE:  *I^2^*, percentage of the total variability due to between-study heterogeneity; P-Heter, P-value of heterogeneity using the Cochran’s Q test.  ^a^ASC and ADHD symptoms within the borderline/clinical range were assessed using parental and teacher reported questionnaires in all cohorts.  ^b^Odds ratios and 95% confidence intervals were estimated by random-effects meta-analysis. Models were adjusted for maternal characteristics (education, age at delivery, pre-pregnancy body mass index, prenatal smoking, mental health during pregnancy, parity and alcohol consumption, fever and infections during pregnancy) and child’s characteristics (sex, age at the behavioral assessment). | | | | | | | | | |

# Table S10. Leave-one-out sensitivity analyses: Adjusted associations between postnatal acetaminophen exposure and autistic spectrum (ASC) symptoms and attention-deficit and hyperactivity (ADHD) symptoms.

|  | **ASC symptoms^a^** | | | |  | **ADHD symptoms^a^** | | | |
| --- | --- | --- | --- | --- | --- | --- | --- | --- | --- |
| **Cohort omitted** | **OR (95 % CI)** | **I2 (%)** | **Q-value** | **P-heter** |  | **OR (95 % CI)** | **I2 (%)** | **Q-value** | **P-heter** |
| **ALSPAC** | 1.05 (0.73, 1.52) | 43.49 | 8.49 | 0.08 |  | 0.99 (0.73, 1.34) | 26.13 | 4.97 | 0.17 |
| **DNBC** | 0.88 (0.62, 1.24) | 27.35 | 6.95 | 0.14 |  | 1.1 (0.79, 1.54) | 6.69 | 3.51 | 0.32 |
| **GASP** | 0.97 (0.69, 1.38) | 58.77 | 11.34 | 0.02 |  | 0.94 (0.74, 1.2) | 7.4 | 2.81 | 0.42 |
| **GenerationR** | 0.91 (0.62, 1.32) | 54.54 | 10.48 | 0.03 |  | 0.87 (0.68, 1.13) | 0 | 2.58 | 0.46 |
| **INMA** | 1.08 (0.79, 1.47) | 41.42 | 8.68 | 0.07 |  | 1.05 (0.73, 1.52) | 31.72 | 4.76 | 0.19 |
| **RHEA** | 1.04 (0.78, 1.4) | 47.91 | 7.39 | 0.12 |  | **-** | **-** | **-** | **-** |
| NOTE:  *I^2^*, percentage of the total variability due to between-study heterogeneity; P-Heter, P-value of heterogeneity using the Cochran’s Q test.  ^a^ASC and ADHD symptoms within the borderline/clinical range were assessed using parental and teacher reported questionnaires in all cohorts.  ^b^Odds ratios and 95% confidence intervals were estimated by random-effects meta-analysis. Models were adjusted for maternal characteristics (education, age at delivery, pre-pregnancy body mass index, prenatal smoking, mental health during pregnancy, parity and alcohol consumption, fever and infections during pregnancy) and child’s characteristics (sex, age at the behavioral assessment, by child’s cold and respiratory infections). | | | | | | | | | |

**References**

Achenbach, T.M, & Rescorla, L. A. (2001). *Manual for the ASEBA School-Age Forms & Profiles*. Burlington, VT: University of Vermont, Research Center for Children, Youth & Families.

Achenbach, Thomas M . (2000). Child Behavior Checklist. In *Encyclopedia of psychology, Vol. 2.* (pp. 69–70). Washington: American Psychological Association. https://doi.org/10.1037/10517-028

Aebi, M., Winkler Metzke, C., & Steinhausen, H.-C. (2010). Accuracy of the DSM-Oriented Attention Problem Scale of the Child Behavior Checklist in Diagnosing Attention-Deficit Hyperactivity Disorder. *Journal of Attention Disorders*, *13*(5), 454–463. https://doi.org/10.1177/1087054708325739

American Psychiatric Association. (2002). *Manual diagnóstico y estadístico de los trastornos mentales [in Spanish]*. Barcelona, Spain: Masson.

Andersen, T. F., Madsen, M., Jørgensen, J., Mellemkjær, L., & Olsen, J. H. (1999). The Danish National Hospital Register: A valuable source of data for modern health sciences. *Danish Medical Bulletin*, *46*(3), 263–268.

Avella-Garcia, C. B., Julvez, J., Fortuny, J., Rebordosa, C., García-Esteban, R., Galán, I. R., … Sunyer, J. (2016). Acetaminophen use in pregnancy and neurodevelopment: attention function and autism spectrum symptoms. *International Journal of Epidemiology*, 1–9. https://doi.org/10.1093/ije/dyw115

Boyd, A., Golding, J., Macleod, J., Lawlor, D. A., Fraser, A., Henderson, J., … Davey Smith, G. (2013). Cohort Profile: The ‘Children of the 90s’—the index offspring of the Avon Longitudinal Study of Parents and Children. *International Journal of Epidemiology*, *42*(1), 111–127. https://doi.org/10.1093/ije/dys064

Chatzi, L., Leventakou, V., Vafeiadi, M., Koutra, K., Roumeliotaki, T., Chalkiadaki, G., … Kogevinas, M. (2017). Cohort Profile: The Mother-Child Cohort in Crete, Greece (Rhea Study). *International Journal of Epidemiology*, *46*(5), 1392-1393k. https://doi.org/10.1093/ije/dyx084

Conners, C. K. (1997). *Conner’s Rating Scales-Revised User’s Manual*. New York: Multi-Health Systems, North Tonawanda.

Foreman, D., Morton, S., & Ford, T. (2009). Exploring the clinical utility of the Development and Well-Being Assessment (DAWBA) in the detection of hyperkinetic disorders and associated diagnoses in clinical practice. *Journal of Child Psychology and Psychiatry and Allied Disciplines*, *50*(4), 460–470. https://doi.org/10.1111/j.1469-7610.2008.02017.x

Fraser, A., Macdonald-wallis, C., Tilling, K., Boyd, A., Golding, J., Davey smith, G., … Lawlor, D. A. (2013). Cohort profile: The avon longitudinal study of parents and children: ALSPAC mothers cohort. *International Journal of Epidemiology*, *42*(1), 97–110. https://doi.org/10.1093/ije/dys066

Golding, J., Gregory, S., Clark, R., Ellis, G., Iles‐Caven, Y., & Northstone, K. (2019). Associations between paracetamol (acetaminophen) intake between 18 and 32 weeks gestation and neurocognitive outcomes in the child: A longitudinal cohort study. *Paediatric and Perinatal Epidemiology*, ppe.12582. https://doi.org/10.1111/ppe.12582

Goodman, R, Ford, T., Richards, H., Gatward, R., & Meltzer, H. (2000). The Development and Well-Being Assessment: description and initial validation of an integrated assessment of child and adolescent psychopathology. *Journal of Child Psychology and Psychiatry, and Allied Disciplines*, *41*(5), 645–655. Retrieved from http://www.ncbi.nlm.nih.gov/entrez/query.fcgi?cmd=Retrieve&db=PubMed&dopt=Citation&list_uids=10946756

Goodman, Robert. (1997). The strengths and difficulties questionnaire: A research note. *Journal of Child Psychology and Psychiatry and Allied Disciplines*, *38*(5), 581–586. https://doi.org/10.1111/j.1469-7610.1997.tb01545.x

Guxens, M., Ballester, F., Espada, M., Fernandez, M. F., Grimalt, J. O., Ibarluzea, J., … Sunyer, J. (2012). Cohort Profile: The INMA--INfancia y Medio Ambiente--(Environment and Childhood) Project. *International Journal of Epidemiology*, *41*(4), 930–940. https://doi.org/10.1093/ije/dyr054

Kooijman, M. N., Kruithof, C. J., van Duijn, C. M., Duijts, L., Franco, O. H., van IJzendoorn, M. H., … Jaddoe, V. W. V. (2016). The Generation R Study: design and cohort update 2017. *European Journal of Epidemiology*, *31*(12), 1243–1264. https://doi.org/10.1007/s10654-016-0224-9

Lee, S. I., Schachar, R. J., Chen, S. X., Ornstein, T. J., Charach, A., Barr, C., & Ickowicz, A. (2008). Predictive validity of DSM-IV and ICD-10 criteria for ADHD and hyperkinetic disorder. *Journal of Child Psychology and Psychiatry*, *49*(1), 70–78. https://doi.org/10.1111/j.1469-7610.2007.01784.x

Liew, Z., Ritz, B., Rebordosa, C., Lee, P.-C., & Olsen, J. (2014). Acetaminophen use during pregnancy, behavioral problems, and hyperkinetic disorders. *JAMA Pediatrics*, *168*(4), 313–320. https://doi.org/10.1001/jamapediatrics.2013.4914

Liew, Z., Ritz, B., Virk, J., & Olsen, J. (2016). Maternal use of acetaminophen during pregnancy and risk of autism spectrum disorders in childhood: A Danish national birth cohort study. *Autism Research : Official Journal of the International Society for Autism Research*, *9*(9), 951–958. https://doi.org/10.1002/aur.1591

Morales-Hidalgo, P., Roigé-Castellví, J., Vigil-Colet, A., & Canals Sans, J. (2017). The Childhood Autism Spectrum Test (CAST): Spanish adaptation and validation. *Autism Research*, *10*(9), 1491–1498. https://doi.org/10.1002/aur.1793

Munk‐Jørgensen, P., Kastrup, M., & Mortensen, P. B. (1993). The Danish psychiatric register as a tool in epidemiology. *Acta Psychiatrica Scandinavica*, *87*(370 S), 27–32. https://doi.org/10.1111/j.1600-0447.1993.tb05358.x

Muratori, F., Narzisi, A., Tancredi, R., Cosenza, A., Calugi, S., Saviozzi, I., … Calderoni, S. (2011). The CBCL 1.5-5 and the identification of preschoolers with autism in Italy. *Epidemiology and Psychiatric Sciences*, *20*(4), 329–338. Retrieved from http://www.ncbi.nlm.nih.gov/pubmed/22201210

Northstone, K., Lewcock, M., Groom, A., Boyd, A., Macleod, J., Timpson, N., & Wells, N. (2019). The Avon Longitudinal Study of Parents and Children (ALSPAC): an update on the enrolled sample of index children in 2019 [version 1; peer review: 2 approved]. *Wellcome Open Research*, *4*, 51. https://doi.org/10.12688/wellcomeopenres.15132.1

Olsen, J., Melbye, M., Olsen, S. F., Sørensen, T. I., Aaby, P., Andersen, A. M., … Søndergaard, C. (2001). The Danish National Birth Cohort--its background, structure and aim. *Scandinavian Journal of Public Health*, *29*(4), 300–307. Retrieved from http://www.ncbi.nlm.nih.gov/pubmed/11775787

Ooi, Y. P., Rescorla, L., Ang, R. P., Woo, B., & Fung, D. S. S. (2011). Identification of Autism Spectrum Disorders using the child behavior checklist in Singapore. *Journal of Autism and Developmental Disorders*, *41*, 1147–1156. https://doi.org/10.1007/s10803-010-1015-x

Porta, D., & Fantini, M. P. (2006). Prospective cohort studies of newborns in Italy to evaluate the role of environmental and genetic characteristics on common childhood disorders. *Italian Journal of Pediatrics*, *32*(6), 350–357. Retrieved from https://www.researchgate.net/publication/289186073_Prospective_cohort_studies_of_newborns_in_Italy_to_evaluate_the_role_of_environmental_and_genetic_characteristics_on_common_childhood_disorders

Russell, G., Rodgers, L. R., & Ford, T. (2013). The Strengths and Difficulties Questionnaire as a Predictor of Parent-Reported Diagnosis of Autism Spectrum Disorder and Attention Deficit Hyperactivity Disorder. *PLoS ONE*, *8*(12), e80247. https://doi.org/10.1371/journal.pone.0080247

Scott, F. J., Baron-Cohen, S., Bolton, P., & Brayne, C. (2002). The CAST (Childhood Asperger Syndrome Test): preliminary development of a UK screen for mainstream primary-school-age children. *Autism*, *6*(1), 9–31. Retrieved from http://www.ncbi.nlm.nih.gov/entrez/query.fcgi?cmd=Retrieve&db=PubMed&dopt=Citation&list_uids=11918111

Skuse, D H, Mandy, W. P., & Scourfield, J. (2005). Measuring autistic traits: heritability, reliability and validity of the Social and Communication Disorders Checklist. *Br J Psychiatry*, *187*, 568–572. Retrieved from http://www.ncbi.nlm.nih.gov/entrez/query.fcgi?cmd=Retrieve&db=PubMed&dopt=Citation&list_uids=16319410

Skuse, David H., Mandy, W., Steer, C., Miller, L. L., Goodman, R., Lawrence, K., … Golding, J. (2009). Social Communication Competence and Functional Adaptation in a General Population of Children: Preliminary Evidence for Sex-by-Verbal IQ Differential Risk. *Journal of the American Academy of Child & Adolescent Psychiatry*, *48*(2), 128–137. https://doi.org/10.1097/CHI.0b013e31819176b8

Snijder, C. A., Kortenkamp, A., Steegers, E. A. P., Jaddoe, V. W. V., Hofman, A., Hass, U., & Burdorf, A. (2012). Intrauterine exposure to mild analgesics during pregnancy and the occurrence of cryptorchidism and hypospadia in the offspring: the Generation R Study. *Human Reproduction*, *27*(4), 1191–1201. https://doi.org/10.1093/humrep/der474

Tick, N. T., Van der Ende, J., Koot, H. M., & Verhulst, F. C. (2007). 14-Year changes in emotional and behavioral problems of very young Dutch children. *Journal of the American Academy of Child and Adolescent Psychiatry*, *46*(10), 1333–1340. https://doi.org/10.1097/chi.0b013e3181337532

Williams, J., Scott, F., Stott, C., Allison, C., Bolton, P., Baron-Cohen, S., & Brayne, C. (2005). The CAST (Childhood Asperger Syndrome Test): Test accuracy. *Autism*, *9*(1), 45–68. https://doi.org/10.1177/1362361305049029
